# Effects of empagliflozin alone and in combination with exercise on the soleus muscle in obese type 2 diabetic rats

**DOI:** 10.1016/j.jphyss.2026.100087

**Published:** 2026-07-07

**Authors:** Kazuho Inoue, Saori Sekiguchi, Yuji Ogura, Seiko Hoshino, Kimie Katayama, Junko Asano, Takayuki Akagi, Junko Igarashi-Migitaka, Shiika Watanabe, Yoshio Nagai, Kenjiro Kimura, Yugo Shibagaki, Atsuko Kamijo-Ikemori

**Affiliations:** a Department of Anatomy, St. Marianna University School of Medicine, Kanagawa, Japan; b Medicine Division, Nippon Boehringer Ingelheim Co., Ltd., Tokyo, Japan; c Division of Nephrology and Hypertension, Department of Internal Medicine, St. Marianna University School of Medicine, Kanagawa, Japan; d Department of Diabetes and Endocrinology, Kanto Rosai Hospital, Kanagawa, Japan; e JCHO Tokyo Takanawa Hospital, Tokyo, Japan; f Institute for Animal Experimentation, St. Marianna University Graduate School of Medicine, Kanagawa, Japan

**Keywords:** Empagliflozin, Exercise, Soleus muscle, Mitochondria, Autophagy

## Abstract

This study assessed whether empagliflozin (EMPA) combined with endurance exercise further improves the soleus muscle in spontaneously diabetic Torii fatty rats, a model of obese type 2 diabetes. Male rats were divided into untreated control, exercise, EMPA, or EMPA + exercise groups. Age-matched Sprague–Dawley rats served as nondiabetic controls. Treadmill training and EMPA (0.03% in chow) were provided from 8 to 16 weeks of age. Both EMPA and the combined intervention improved blood glucose and insulin resistance. Additionally, the combined treatment reduced total cholesterol and triglycerides compared with EMPA alone. The cross-sectional area of type I fibers in the soleus tended to improve with all interventions. Combination treatment significantly enhanced citrate synthase activity, mitochondrial functional protein expression, and autophagic flux. These findings suggest that although EMPA does not adversely affect the soleus muscle, combining it with endurance exercise confers superior benefits via improved mitochondrial status, autophagy, and lipid metabolism.

## Highlights

• Empagliflozin plus exercise improves soleus muscle in obese type 2 diabetes.
• Combined treatment enhances mitochondrial status in diabetic soleus muscle.
• Combined treatment promotes autophagy and preserves type I fiber size.
• Empagliflozin alone does not reduce muscle mass in diabetes.

## Introduction

A progressive decline in muscle mass and strength, hallmarks of sarcopenia, is associated with reduced social independence and shortened healthy life expectancy [1]. The prevalence of obesity-related type 2 diabetes mellitus (T2DM) has increased with lifestyle changes, and affected patients are at high risk of sarcopenia [2], [3]. Therefore, T2DM management should be guided by careful consideration of patients’ quality of life and activities of daily living.

Sodium-glucose cotransporter 2 (SGLT2) inhibitors prevent glucose reabsorption in the renal proximal tubules by blocking SGLT2, thereby promoting urinary glucose excretion and reducing blood glucose levels. These agents are widely used as antihyperglycemic agents. However, because SGLT2 inhibitors have been reported to decrease body weight accompanied by reductions in skeletal muscle mass in clinical practice [4], there is concern that they may exacerbate sarcopenia in obese T2DM patients with high risk of sarcopenic obesity.

In contrast, a recent clinical trial demonstrated that empagliflozin (EMPA), an SGLT2 inhibitor, did not aggravate the loss of muscle mass or strength in elderly patients with T2DM over 65 years old [5]. Furthermore, an experimental study showed that SGLT2 inhibitors attenuated skeletal muscle atrophy in obese T2DM mice [6]. Additionally, the combination of an SGLT2 inhibitor and exercise has been reported to enhance the exercise capacity in mice with type 1 diabetes [7]. These findings suggest that SGLT2 inhibitors may exert beneficial effects on skeletal muscle, either as a standalone intervention or when combined with exercise.

In humans, obesity-associated T2DM is characterized by hyperglycemia and comorbidities, including hypertension and dyslipidemia. Under these conditions, type I (slow-twitch) muscle fibers are particularly susceptible to dysfunction [8], [9]. This muscle dysfunction has been linked to mitochondrial dysfunction and dysregulated autophagy [10], [11]. Furthermore, previous studies have demonstrated that SGLT2 inhibitors activate autophagy-related signaling pathways in various organs [12], [13], [14]. However, the effects of SGLT2 inhibitors, with or without exercise, on mitochondrial profile and autophagy-related processes in slow-twitch muscles of obese T2DM with metabolic complications remain unclear. Therefore, this study aimed to investigate the effects of an SGLT2 inhibitor, both alone and in conjunction with exercise, on mitochondrial markers (citrate synthase activity and protein expression) and autophagy-related processes in the soleus muscle—which predominantly consists of slow-twitch muscle fibers—in male spontaneously diabetic Torii (SDT) fatty rats (SDT.Cg-Leprfa/JttJcl), an established animal model of obesity, T2DM, and their associated metabolic complications.

## Materials and methods

### Animals

All animal experiments were performed following the ethical standards of St. Marianna University School of Medicine. The experimental protocol was approved by the Animal Experiment Committee at the Institute for Animal Experimentation, St. Marianna University Graduate School of Medicine (Approval No. 2406001).

5-week-old male SDT fatty rats (n = 29) and age-matched Sprague–Dawley (SD) rats (n = 8) were purchased from CLEA Japan (Tokyo, Japan). Both strains are commercially available laboratory animals. SDT fatty rats are a well-established model of obesity-associated T2DM, exhibiting early-onset hyperglycemia, insulin resistance, and progressive skeletal muscle atrophy [15], [16], [17], [18], [19], [20], [21], [22], [23]. SD rats, derived from the same genetic background as the original SDT strain, were used as nondiabetic controls to establish a baseline to assess diabetes-induced alterations.

All animals were housed at the Institute for Animal Experimentation, St. Marianna University School of Medicine, under controlled conditions (24°C, 12-h light/dark cycle) with open access to standard laboratory chow (CE−2; CLEA Japan) and water.

### Experimental design

8-week-old SDT fatty rats were randomly assigned to the untreated control group (SDT-Cont, n = 8), the exercise group (SDT-Ex, n = 7), the EMPA treatment group (SDT-EMPA, n = 8), or the EMPA plus exercise treatment group (SDT-EMPA + Ex, n = 6). Additionally, age-matched SD rats served as the untreated nondiabetic control group (n = 8).

The SDT-Ex and SDT-EMPA + Ex groups participated in a treadmill-based endurance training program (KN−73 TREADMILL; Natsume Seisakusho, Tokyo, Japan) four times per week for 8 weeks, with the intensity gradually increased. The training regimen, starting at 8 weeks of age and continuing for 8 weeks, was as follows: from 8 to 10 weeks, rats performed a 5-min warm-up at 7–10 m/min, followed by running at 10–12 m/min, with the exercise duration increased by 10 min each week (i.e., 10 min at 8 weeks, 20 min at 9 weeks, and 30 min at 10 weeks). During 11–16 weeks, a warm-up was performed at 10 m/min for 5 min, followed by running at 13–20 m/min for 30 min. The treadmill inclination was set to 3° throughout the training period (Table 1). Previous studies have defined treadmill running at approximately 15–20 m/min in rats as moderate-intensity aerobic exercise [24]. Thus, the running speed used in our protocol corresponds to moderate-intensity endurance exercise. The exercise duration was set according to our previous study in SDT fatty rats [22], [23], taking feasibility and tolerability into account.

**Table 1:** Details of the training regimen for the Ex and EMPA + Ex group at 8–16 weeks.

**Age**	**Warm-up**	**Exercise**	**Inclination**
8w	7–8 m/min, 5 min	10 m/min, 10 min	3°
9w	7–10 m/min, 5 min	11–12 m/min, 20 min	3°
10w	8–10 m/min, 5 min	12 m/min, 30 min	3°
11w	10 m/min, 5 min	13–14 m/min, 30 min	3°
12w	10 m/min, 5 min	15 m/min, 30 min	3°
13w	10 m/min, 5 min	17–18 m/min, 30 min	3°
14w	10 m/min, 5 min	19.5–20 m/min, 30 min	3°
15w	10 m/min, 5 min	18–20 m/min, 30 min	3°
16w	10 m/min, 5 min	18 m/min, 30 min	3°

The SDT-EMPA and SDT-EMPA + Ex groups received EMPA-mixed chow ad libitum from 8 to 16 weeks old. EMPA was provided by Boehringer Ingelheim (Ingelheim, Germany) under a collaborative research agreement. It was incorporated into a diet identical in composition to that of the untreated rats at a concentration of 0.03% and administered to the animals as a solid diet.

Body weight, food intake, water intake, grip strength, and blood glucose levels were measured every 4 weeks beginning at 8 weeks of age. Muscle strength was assessed using a grip strength meter (MK−380CM/FM; Muromachi Kikai, Tokyo, Japan) with a four-limb grip test. The results were averaged across three attempts per rat, and the values were normalized to body weight (kg).

At 16 weeks of age, after completing all interventions, the rats were anesthetized with isoflurane, and blood was collected from the inferior vena cava. After exsanguination and euthanasia, peritesticular and perirenal adipose tissues, and the soleus muscles from both hindlimbs, were excised and weighed. Adipose tissues were solely used for weight measurements and were not subjected to further analyses. The left soleus muscle was collected for molecular analyses, and the right soleus muscle was collected for histological analyses. Each muscle was immediately frozen in liquid nitrogen and stored at −80 °C until analysis. After muscle sampling, the tibial length from both hindlimbs was measured with a digital caliper.

### Measurement of blood glucose, serum insulin, and lipid parameters

To longitudinally monitor blood glucose levels, the rats were anesthetized with isoflurane, and a small incision was made at the tail tip with a razor blade. Blood glucose was measured with a portable glucose meter (FreeStyle Freedom Lite; Nipro, Osaka, Japan) and compatible glucose test strips (Nipro FS Blood Glucose Sensor Lite; Nipro), and the results are expressed as mg/dL. For fasting blood glucose measurements, the rats were fasted for 16 h before sacrifice, and blood samples collected at sacrifice were used for analysis. At sacrifice, blood was collected from the inferior vena cava, and serum was obtained via centrifugation. Serum insulin concentrations were measured using a rat insulin ELISA kit (Levis Rat Insulin ELISA Kit; Fujifilm Wako Pure Chemical Corporation, Osaka, Japan), and the results are expressed as µIU/mL. Insulin resistance was evaluated using the Homeostasis Model Assessment of Insulin Resistance (HOMA-IR), calculated as follows: fasting blood glucose (mg/dL) × fasting serum insulin (µIU/mL)/405. Total cholesterol and triglyceride levels were measured using an external laboratory service (SRL, Tokyo, Japan).

### Fluorescent immunohistochemical analysis of soleus muscle tissue

Frozen soleus muscle samples were sectioned into 10-µm slices with a cryostat (HM 550; Thermo Fisher Scientific, Rockford, IL, USA). To assess the cross-sectional area (CSA) of the type I muscle fibers, double-label fluorescent immunohistochemistry was performed. The sections were incubated overnight at 4°C with primary antibodies against myosin heavy chain (MyHC) isoforms: anti-type I MyHC IgG2b (1:200; BA-F8; Developmental Studies Hybridoma Bank, University of Iowa, Iowa City, IA, USA), anti-type IIb MyHC IgG1 (1:100; F18; Developmental Studies Hybridoma Bank), and anti-laminin IgG2a (1:200; 2E8; Developmental Studies Hybridoma Bank). The sections were then incubated with fluorescent secondary antibodies: Alexa Fluor 488-labeled goat antimouse IgG2b (1:500; A−21141; Invitrogen, Carlsbad, CA, USA), Alexa Fluor 568-labeled goat antimouse IgG1 (1:500; A−21124; Invitrogen), and Alexa Fluor 488-labeled goat antimouse IgG2a (1:500; A−21131; Invitrogen). CSA was measured with WinROOF software (Mitani Corporation, Tokyo, Japan). For each sample, CSA values were calculated from 300 type I fibers, and the average was used for subsequent analysis.

### Citrate synthase activity

Citrate synthase (CS) activity was measured as an index of mitochondrial content in the soleus muscle according to a previously reported method [21], [25]. Frozen soleus muscle samples were homogenized in T-PER Tissue Protein Extraction Reagent (Thermo Fisher Scientific) supplemented with protease and phosphatase inhibitors. Following centrifugation at 15,000 rpm for 10 min to remove debris, the supernatants were collected, and protein concentrations were determined with the Bradford assay (Bio-Rad). The reaction mixture contained 100 mM Tris buffer, 0.07% Triton X−100, 0.1 mM Ellman’s reagent, 0.098 mM acetyl-CoA, and 0.5 mM oxaloacetic acid (pH 8.3). An appropriate volume of muscle supernatant was added to initiate the reaction. CS activity was determined spectrophotometrically by monitoring the increase in absorbance at 412 nm with a microplate reader (SpectraMax 190 EXT; Molecular Devices Japan, Tokyo, Japan). Each sample was measured in duplicate, and background absorbance from blank reactions was subtracted. Activity was calculated from the slope of the absorbance change over time during the most linear portion of the reaction curve and is expressed as specific activity normalized to protein concentration (mM·min⁻¹·mg protein⁻¹).

### Western blotting

Proteins extracted from the soleus muscle (15 µg per well) were separated on NuPAGE 4%–12% Bis-Tris gels (Thermo Fisher Scientific) and transferred onto a polyvinylidene fluoride membrane using HorizeBLOT 4 M and EzFastBlot HMW (ATTO, Tokyo, Japan). Before incubation with primary antibodies, the membranes were cut near the molecular weights of each target protein. The following primary antibodies were used: Peroxisome proliferator-activated receptor gamma coactivator 1-α (PGC−1α, 1:1000; ab191838; Abcam, Cambridge, UK), medium-chain specific acyl-CoA dehydrogenase (MCAD, 1:10,000; ab92461; Abcam), cytochrome c oxidase subunit 5B (COX5B, 1:10,000; ab180136; Abcam), microtubule-associated protein 1 light chain 3 beta (LC3B, 1:1000; #2775; Cell Signaling Technology, Danvers, MA, USA), phospho-Unc−51-like kinase 1 (Ser317) (p-ULK1, 1:1000; #89267; Cell Signaling Technology), ULK1 (1:1000; #8054; Cell Signaling Technology), phospho-sequestosome 1 (SQSTM1/p62) (Ser403) (p-p62, 1:1000; #39786; Cell Signaling Technology), p62 (1:1000; #23214; Cell Signaling Technology), and α-tubulin (1:8000; ab176560; Abcam). For the rabbit primary antibodies, the secondary antibody was Rabbit IgG H&L (1:20,000; ab97051; Abcam). Immunoreactive bands were detected with ECL Prime Western Blotting Detection Reagent (GE Healthcare, Little Chalfont, UK). Protein expression levels were quantified with ImageJ software (National Institutes of Health, Bethesda, MD, USA) and normalized to the level of α-tubulin. For LC3B, the ratio of LC3B-II to LC3B-I was calculated. Phosphorylated ULK1 and p62 were normalized to their respective total protein levels. The entire Western blot images corresponding to the results presented in Fig. 5, Fig. 6, Fig. 7 are provided in the Supplementary Data (Supplementary Figs. S2–S4).

### Statistical analysis

Continuous variable distributions were assessed with the Shapiro–Wilk test, and normality test results are provided in Supplementary Table S1. All data are presented as medians with ranges. For variables that were normally distributed, parametric statistical analyses were applied, and the corresponding data are presented as means ± standard error of the mean in Supplementary Table S2. For comparisons among the five groups, normally distributed data were analyzed with one-way analysis of variance followed by Tukey’s honestly significant difference test. Nonnormally distributed data were analyzed with the Kruskal–Wallis test followed by the Steel–Dwass post-hoc test. For longitudinal data collected at multiple time points, differences over time were analyzed using the Friedman rank test. When a significant main effect was detected, post-hoc multiple comparisons were performed using the Nemenyi test. All statistical analyses were conducted in JMP software version 18 (SAS Institute, Cary, NC, USA). P-values < 0.05 were considered statistically significant.

## Results

### Changes in body weight, food and water intake, grip strength, and blood glucose level

Body weight increased with age in all groups from 8 to 16 weeks old. At 8 weeks, body weight was significantly higher in all SDT groups than in the SD group. At 12 weeks, the SDT-EMPA group had even higher body weights compared with the SD, SDT-Cont, and SDT-Ex groups. However, no significant differences were observed among the remaining SDT groups. At 16 weeks, the SDT-EMPA group had the highest body weight, which was significantly greater than that of the SD, SDT-Cont, and SDT-Ex groups. The SDT-EMPA + Ex group also demonstrated a significant increase in body weight compared with the SDT-Ex group (Table 2).

**Table 2:** Time-related changes in various parameters.

Parameter	Group	8 weeks of age	12 weeks of age	16 weeks of age
Body weight (g)	SD	279.3 (266.3–326.6)	484.6 (422.5–539.1)	539.9 (482.7–595.6)^‡‡^
	SDT-Cont	365.5 (349.1–403.5) **	520.3 (465.3–558.4)	559.6 (494.7–634.1)^‡‡^
	SDT-Ex	369.7 (356.9–379.0) *	492.4 (471.2–528.3)	517.5 (505.5–554.4)^‡‡^
	SDT-EMPA	370.6 (322.8–394.8) *	578.8 (543.6–606.3) **^, #, †^	677.4 (621.9–696.4) **^, #, †, ‡‡^
	SDT-EMPA + Ex	365.7 (325.7–376.6) *	546.0 (500.7–578.2)	616.3 (559.8–660.9)^†, ‡‡^
Food intake (g)	SD	30.9 (28.9–34.4)	28.5 (24.3–30.2)	24.4 (21.1–31.7)^‡^
	SDT-Cont	50.3 (39.5–54.8) **	56.6 (40.3–59.5) **^, ‡‡^	55.9 (40.9–66.7) **^, ‡^
	SDT-Ex	52.4 (43.8–55.6) *	58.4 (42.5–69.3) *	60.3 (47.5–73.6) *^, ‡^
	SDT-EMPA	51.4 (46.2–55.8) **	55.4 (46.3–65.2) **^, ‡^	54.4 (46.3–63.4) **
	SDT-EMPA + Ex	52.6 (47.5–59.4) *	56.0 (43.8–59.6) *	53.1 (45.5–62.6) *
Water intake (g)	SD	5.9 (2.0–18.7)	13.0 (0.5–21.4)	11.8 (0.0–36.6)
	SDT-Cont	5.0 (1.3–21.8)	15.8 (0.4–32.3)	17.5 (0.9–31.8)
	SDT-Ex	7.0 (2.4–25.8)	11.5 (5.1–26.1)	20.6 (10.9–32.3)^‡^
	SDT-EMPA	5.3 (0.7–19.1)	21.3 (17–26.5)^‡^	24.4 (11.8–40.9)^‡‡^
	SDT-EMPA + Ex	6.7 (2.4–14.9)	11.3 (7.2–18.3)	11.3 (4.3–17.1)
Grip strength/ body weight (N/kg)	SD	58.0 (51.9–67.0)	62.8 (50.9–75.1)	53.5 (47.4–64.2)
	SDT-Cont	38.3 (34.1–43.3) **	41.6 (36.1–43.7) **	40.5 (32.3–50.7) *
	SDT-Ex	38.8 (34.6–42.4) *	40.4 (39.0–48.4) *	42.9 (33.8–47.1) *
	SDT-EMPA	39.6 (27.9–44.0) **	40.2 (36.4–43.8) **	39.5 (35.0–43.2) **
	SDT-EMPA + Ex	36.0 (31.9–42.1) *	42.6 (37.5–51.5) *	36.1 (30.9–40.0) *
Blood glucose level (mg/dL)	SD	91 (78–117)	97 (81–124)	96 (76–115)
	SDT-Cont	301 (162–500) **	404 (299–470) **	456 (377–500) **^, ‡^
	SDT-Ex	334 (259–500) *	500 (373–500) *	439 (362–500) *
	SDT-EMPA	303 (178–479) **	123 (105–181)^##, †, ‡‡^	149 (113–180) *^, ##, †, ‡‡^
	SDT-EMPA + Ex	405 (288–500) *	181 (132–214) *^, #, †^	157 (96–259)^#, †, ‡‡^

All data are presented as medians with ranges. Non-normally distributed data were analyzed using the Kruskal–Wallis test followed by the Steel–Dwass post hoc test. For longitudinal comparisons within the same group across multiple time points, differences over time were analyzed using the Friedman rank test, followed by the Nemenyi post hoc test when a significant main effect was detected. Rats were divided into five groups: a non-diabetic untreated control group (SD, n = 8), an untreated control group (SDT-Cont, n = 8), an exercise group (SDT-Ex, n = 7), an empagliflozin treatment group (SDT-EMPA, n = 8), and an exercise plus empagliflozin treatment group (SDT-EMPA+Ex, n = 6).* *p* < 0.05 and ** *p* < 0.01 vs. SD group at the same age; ^#^*p* < 0.05 and ^##^*p* < 0.01 vs. SDT-Cont group at the same age; ^†^*p* < 0.05 vs. SDT-Ex group at the same age; ^‡^*p* < 0.05 and ^‡‡^*p* < 0.01 vs. the same group at 8 weeks old.

Food intake was consistently higher in all SDT groups than in the SD group across all time points. At 12 weeks, the SDT-Cont and SDT-EMPA groups had higher values than at 8 weeks. At 16 weeks, food intake was lower in the SD group than at 8 weeks of age, whereas　the SDT-Cont and SDT-Ex groups showed higher intake than at 8 weeks of age. No significant differences were observed among the SDT groups (Table 2).

Water intake did not differ significantly among the groups at 8 weeks. At 12 and 16 weeks, water intake was significantly higher in the SDT-EMPA group compared with that at 8 weeks. Additionally, water intake at 16 weeks was significantly higher than at 8 weeks in the SDT-Ex group (Table 2).

Grip strength normalized to body weight was significantly lower in all SDT groups than in the SD group at all ages. No significant age-dependent changes in grip strength were observed within any group (Table 2).

Blood glucose levels were markedly elevated in all SDT groups compared with the SD group at 8 weeks. At 12 and 16 weeks, blood glucose levels remained significantly higher in the SDT-Cont and SDT-Ex groups than in the SD group. Furthermore, blood glucose levels in the SDT-Cont group increased significantly by 16 weeks compared with 8 weeks. Conversely, the two EMPA-treated groups had significantly lower blood glucose levels at 12 and 16 weeks than the SDT-Cont and SDT-Ex groups. Although the EMPA group showed no significant differences compared with the SD group at 12 weeks, it exhibited significantly higher levels at 16 weeks. The EMPA + Ex group showed significantly higher blood glucose levels than the SD group at 12 weeks, but there was no significant difference at 16 weeks. Within-group comparisons relative to 8 weeks demonstrated significant reductions in blood glucose levels in the SDT-EMPA group at both 12 and 16 weeks and in the SDT-EMPA + Ex group at 16 weeks (Table 2).

### Comparison of fasting blood glucose level and serum biochemistry

At 16 weeks, fasting blood glucose levels were significantly higher in the SDT-Cont, SDT-Ex, and SDT-EMPA + Ex groups than in the SD group. Fasting blood glucose levels were significantly lower in the SDT-Ex, SDT-EMPA, and SDT-EMPA + Ex groups than in the SDT-Cont group. Furthermore, fasting blood glucose in the SDT-EMPA and SDT-EMPA + Ex groups was significantly lower than that in the SDT-Ex group (Table 3). Fasting serum insulin levels were markedly elevated in all SDT groups compared to those in the SD group. Conversely, the SDT-EMPA + Ex group demonstrated a significant decrease compared to the SDT-Cont group (Table 3). Consistent with changes in fasting glucose and insulin levels, HOMA-IR was significantly increased in all SDT groups compared with the SD group. However, HOMA-IR was significantly lower in the SDT-EMPA and SDT-EMPA + Ex groups than in the SDT-Cont group, whereas the SDT-Ex group did not differ significantly from the SDT-Cont group (Table 3).

**Table 3:** Fasting blood glucose, fasting serum insulin, HOMA-IR, total cholesterol, and triglyceride levels at 16 weeks of age.

Parameter	Group	16 weeks of age
Fasting blood glucose level (mg/dL)	SD	98 (75–127)
	SDT-Cont	265 (212–434) **
	SDT-Ex	182 (160–222) *^, #^
	SDT-EMPA	125 (99–166)^##, †^
	SDT-EMPA + Ex	143 (118–161) *^, #, †^
Fasting serum insulin level (µIU/mL)	SD	4.8 (3.4–9.7)
	SDT-Cont	26.9 (21.6–125.6) **
	SDT-Ex	20.6 (12.5–101.2) *
	SDT-EMPA	17.7 (5.5–33.7) *
	SDT-EMPA + Ex	16.3 (8.0–20.4) *^, #^
HOMA-IR	SD	1.34 (0.63–2.45)
	SDT-Cont	19.87 (13.25–66.98) **
	SDT-Ex	8.73 (5.61–45.49) *
	SDT-EMPA	4.81 (1.35–13.01) *^, ##^
	SDT-EMPA + Ex	6.08 (2.96–6.64) *^, #^
Total cholesterol level (mg/dL)	SD	69 (51–79)
	SDT-Cont	155 (124–200) **
	SDT-Ex	135 (115–180) **
	SDT-EMPA	175 (140–192) **^, †^
	SDT-EMPA + Ex	129 (112–165) **^, §^
Triglyceride level (mg/dL)	SD	43 (25–109)
	SDT-Cont	599 (452–681) **
	SDT-Ex	366 (272–506) *^, #^
	SDT-EMPA	635 (434–776) **^, †^
	SDT-EMPA + Ex	367 (258–442) *^, #, §^

All data are presented as medians with ranges. Non-normally distributed data, except for total cholesterol levels, were analyzed using the Kruskal–Wallis test followed by the Steel–Dwass post hoc test, whereas normally distributed total cholesterol levels were analyzed using one-way ANOVA followed by Tukey’s post hoc test. Although parametric analyses were applied, total cholesterol data are presented as medians in the figure to align with the data reporting in the manuscript, and mean ± standard error of the mean (SEM) values for normally distributed data are provided in Supplementary Table S2. Rats were divided into five groups: a non-diabetic untreated control group (SD, n = 8), an untreated control group (SDT-Cont, n = 8), an exercise group (SDT-Ex, n = 7), an empagliflozin treatment group (SDT-EMPA, n = 8), and an exercise plus empagliflozin treatment group (SDT-EMPA+Ex, n = 6). * *p* < 0.05 and ** *p* < 0.01 vs. SD group; ^#^*p* < 0.05 and ^##^*p* < 0.01 vs. SDT-Cont group; ^†^*p* < 0.05 vs. SDT-Ex group; ^§^*p* < 0.05 vs. SDT-EMPA group.

Total cholesterol was significantly higher in all SDT groups than in the SD group. Total cholesterol levels were significantly higher in the SDT-EMPA group than in the SDT-Ex group, whereas the SDT-EMPA + Ex group demonstrated a significant reduction compared with the SDT-EMPA group (Table 3). Triglyceride levels were also markedly higher in all SDT groups compared with the SD group. Triglyceride levels were significantly reduced in the SDT-Ex and SDT-EMPA + Ex groups compared with the SDT-Cont group. Conversely, the SDT-EMPA group showed significantly higher triglyceride levels than the SDT-Ex group, whereas the SDT-EMPA + Ex group had significantly lower levels than the SDT-EMPA group (Table 3).

### Comparison of adipose tissue weight

**Fig. 1:**
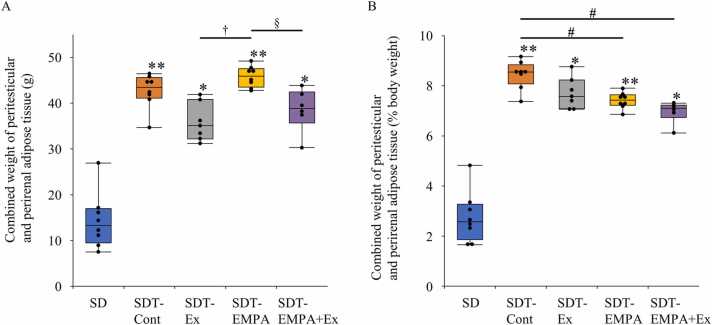
**Comparison of peritesticular and perirenal adipose tissue weights.** Combined weight of peritesticular and perirenal adipose tissue (g) (A). Combined weight of peritesticular and perirenal adipose tissue expressed as a percentage of body weight (% body weight) (B). Rats were divided into five groups: a non-diabetic untreated control group (SD, n = 8), an untreated control group (SDT-Cont, n = 8), an exercise group (SDT-Ex, n = 7), an empagliflozin treatment group (SDT-EMPA, n = 8), and an exercise plus empagliflozin treatment group (SDT-EMPA+Ex, n = 6). Data are presented as box-and-whisker plots, showing the median, interquartile range, and minimum and maximum values, with individual data points overlaid. Non-normally distributed data were analyzed using the Kruskal–Wallis test followed by the Steel–Dwass post hoc test. * *p* < 0.05 and ** *p* < 0.01 vs. SD group; ^#^*p* < 0.05 vs. SDT-Cont group; ^†^*p* < 0.05 vs. SDT-Ex group; ^§^*p*^<^ 0.05 vs. SDT-EMPA group.

The combined weight of the peritesticular and perirenal adipose tissue was significantly higher in all SDT groups than in the SD group. Among the SDT groups, the SDT-EMPA group exhibited a significantly higher combined adipose tissue weight than the SDT-Ex and SDT-EMPA + Ex groups (Fig. 1A). When the combined weight of the peritesticular and perirenal adipose tissue was normalized to body weight, all SDT groups showed significantly higher values than the SD group. In contrast, body weight normalized adipose tissue weight was significantly lower in the SDT-EMPA and SDT-EMPA + Ex groups than in the SDT-Cont group. No significant differences were observed between the SDT-Ex and SDT-Cont groups (Fig. 1B).

### Comparison of soleus muscle weight and CSA

**Fig. 2:**
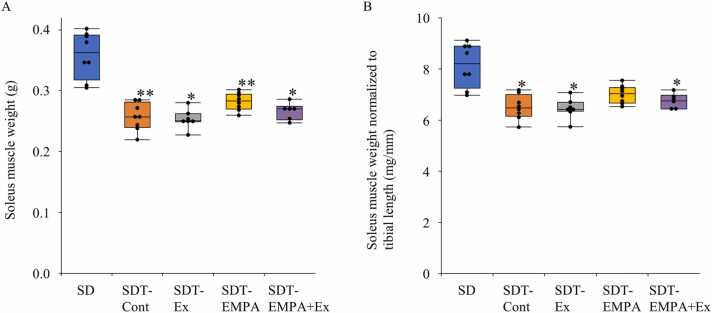
**Comparison of soleus muscle weight.** Absolute soleus muscle weight (g) (A). Soleus muscle weight normalized to tibial length (mg/mm) (B), which was used as an index of body size to avoid potential bias caused by significant differences in body weight among groups. Rats were divided into five groups: a non-diabetic untreated control group (SD, n = 8), an untreated control group (SDT-Cont, n = 8), an exercise group (SDT-Ex, n = 7), an empagliflozin treatment group (SDT-EMPA, n = 8), and an exercise plus empagliflozin treatment group (SDT-EMPA+Ex, n = 6). Data are presented as box-and-whisker plots showing the median, interquartile range, and minimum and maximum values, with individual data points overlaid. Non-normally distributed data were analyzed using the Kruskal–Wallis test followed by the Steel–Dwass post hoc test. * *p* < 0.05 and ** *p* < 0.01 vs. SD group.

Soleus muscle weight was significantly lower in all SDT groups than in the SD group, with no significant differences observed among the SDT groups (Fig. 2A). Soleus muscle weight normalized to tibial length was also evaluated. The tibial length–normalized soleus muscle weight was significantly lower in the SDT-Cont, SDT-Ex, and SDT-EMPA + Ex groups compared with the SD group. However, no significant differences were observed between the SD and SDT-EMPA groups. No significant differences were detected among the SDT groups (Fig. 2B).

**Fig. 3:**
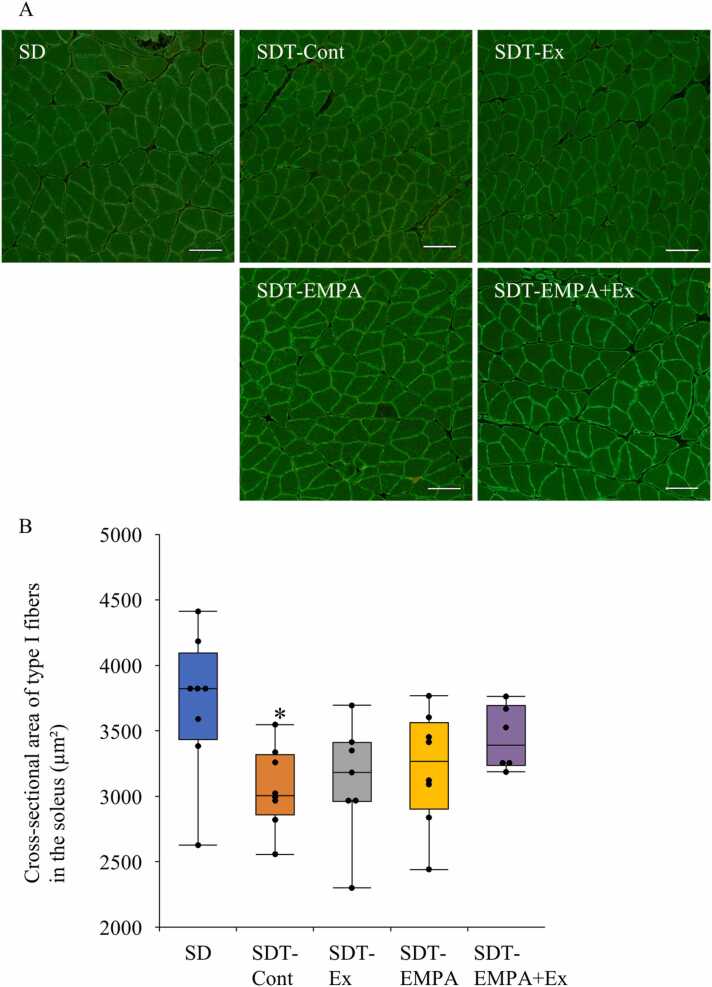
**Comparison of the cross-sectional area of type I fibers in the soleus muscle.** Representative images of type I fibers (green) in the soleus muscle (A). Quantification of the cross-sectional area (CSA, µm²) of type I fibers (B). Rats were divided into five groups: a non-diabetic untreated control group (SD, n = 8), an untreated control group (SDT-Cont, n = 8), an exercise group (SDT-Ex, n = 7), an empagliflozin treatment group (SDT-EMPA, n = 8), and an exercise plus empagliflozin treatment group (SDT-EMPA+Ex, n = 6). Data are presented as box-and-whisker plots, showing the median, interquartile range, and minimum and maximum values, with individual data points overlaid. Because CSA data were normally distributed, parametric statistical analyses were applied using one-way analysis of variance (ANOVA) followed by Tukey’s honestly significant difference (HSD) post hoc test. Although parametric analyses were applied, CSA data are presented as medians in the figure to align with the data reporting in the manuscript, and mean ± standard error of the mean (SEM) values for normally distributed data are provided in Supplementary Table S2. * *p* < 0.05 vs. SD group. Scale bars = 100 µm.

The CSA of type I muscle fibers in the soleus did not differ significantly among the SDT groups. Compared with the SD group, a significant reduction in type I fiber CSA was observed only in the SDT-Cont group, whereas no significant differences were found in the other SDT groups (Fig. 3A, B).

### Analysis of mitochondrial content and functional protein expression in the soleus muscle

**Fig. 4:**
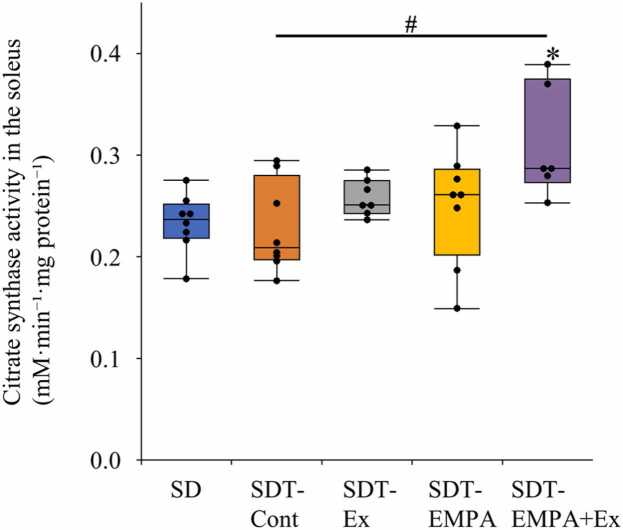
**Assessment of citrate synthase activity in the soleus muscle.** Citrate synthase activity (CS activity, mM·min⁻¹·mg protein⁻¹) in the soleus muscle. Rats were divided into five groups: a non-diabetic untreated control group (SD, n = 8), an untreated control group (SDT-Cont, n = 8), an exercise group (SDT-Ex, n = 7), an empagliflozin treatment group (SDT-EMPA, n = 8), and an exercise plus empagliflozin treatment group (SDT-EMPA+Ex, n = 6). Data are presented as box-and-whisker plots, showing the median, interquartile range, and minimum and maximum values, with individual data points overlaid. Because CS activity data were normally distributed, parametric statistical analyses were applied using one-way analysis of variance (ANOVA) followed by Tukey’s honestly significant difference (HSD) post hoc test. Although parametric analyses were applied, CS activity data are presented as medians in the figure to align with the data reporting in the manuscript, and mean ± standard error of the mean (SEM) values for normally distributed data are provided in Supplementary Table S2. * *p* < 0.05 vs. SD group; ^#^*p* < 0.05 vs. SDT-Cont group.

CS activity, an index of mitochondrial content, was not significantly different among the SD, SDT-Cont, SDT-Ex, and SDT-EMPA groups. Conversely, CS activity in the SDT-EMPA + Ex group was significantly higher than in both the SD and SDT-Cont groups (Fig. 4).

**Fig. 5:**
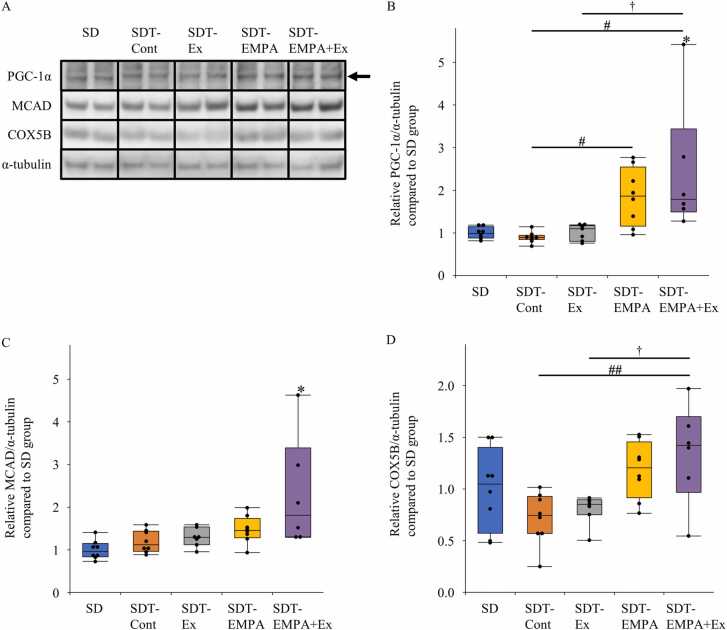
**Quantification of PGC−1α, MCAD, and COX5B in the soleus muscle by Western blotting.** Representative Western blotting bands of PGC−1α, MCAD, COX5B, and α-tubulin in each group on the same membrane (A). The arrow indicates the target PGC−1α band. Samples from the same experiment were processed in parallel for SDS polyacrylamide gel electrophoresis (SDS-PAGE) and Western blotting using different gels and membranes, and the image data obtained were cropped. Entire Western blot images are shown in Supplementary Figs. S2. Quantification of protein expression relative to α-tubulin and normalized to the SD group: PGC−1α/α-tubulin (B), MCAD/α-tubulin (C), and COX5B/α-tubulin (D). Rats were divided into five groups: a non-diabetic untreated control group (SD, n = 8), an untreated control group (SDT-Cont, n = 8), an exercise group (SDT-Ex, n = 7), an empagliflozin treatment group (SDT-EMPA, n = 8), and an exercise plus empagliflozin treatment group (SDT-EMPA+Ex, n = 6). Data are presented as box-and-whisker plots, showing the median, interquartile range, and minimum and maximum values, with individual data points overlaid. Non-normally distributed data (PGC−1α/α-tubulin and MCAD/α-tubulin) were analyzed using the Kruskal–Wallis test followed by the Steel–Dwass post hoc test, whereas normally distributed data (COX5B/α-tubulin) were analyzed using one-way ANOVA followed by Tukey’s post hoc test. Although parametric analyses were applied, COX5B/α-tubulin data are presented as medians in the figure to align with the data reporting in the manuscript, and mean ± standard error of the mean (SEM) values for normally distributed data are provided in Supplementary Table S2. * *p* < 0.05 vs. SD group; ^#^*p* < 0.05 and ^##^*p* < 0.01 vs. SDT-Cont group; ^†^*p* < 0.05 vs. SDT-Ex group.

Expression of the mitochondrial biogenesis-related protein PGC−1α was significantly higher in the SDT-EMPA group than in the SDT-Cont group. The SDT-EMPA + Ex group showed significantly elevated expression compared with the SD, SDT-Cont, and SDT-Ex groups, whereas no significant differences were observed among the other groups (Fig. 5A, B). For the fatty acid β-oxidation–related enzyme MCAD, a significant increase was observed only in the SDT-EMPA + Ex group compared with the SD group, with no other significant differences among the groups (Fig. 5A, C). Expression of the electron transport chain–related enzyme COX5B was significantly higher in the SDT-EMPA + Ex group than in the SDT-Cont and SDT-Ex groups. No other significant differences were observed (Fig. 5A, D).

### Analysis of autophagy-related molecules in the soleus muscle

**Fig. 6:**
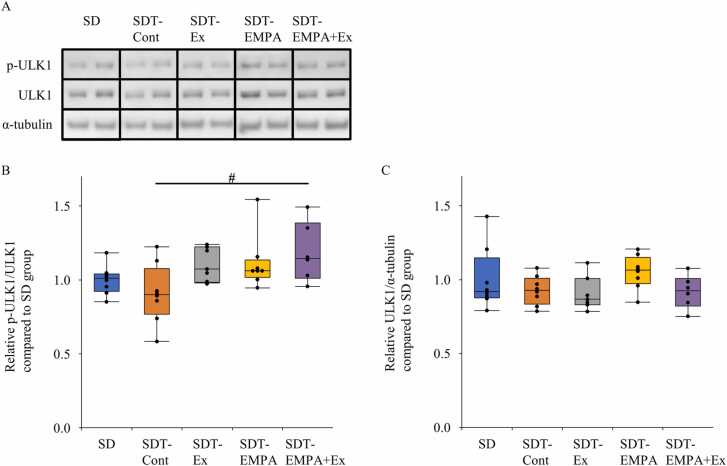
**Quantification of ULK1 phosphorylation in the soleus muscle by Western blotting.** Representative Western blotting bands of phosphorylated ULK1 (p-ULK1), total ULK1 (ULK1), and α-tubulin in each group on the same membrane (A). Samples from the same experiment were processed in parallel for SDS polyacrylamide gel electrophoresis (SDS-PAGE) and Western blotting using different gels and membranes, and the image data obtained were cropped. Entire Western blot images are shown in Supplementary Figs. S3. Quantification of protein expression: relative p-ULK1/ULK1 (B) and relative ULK1/α-tubulin (C), both normalized to the SD group. Rats were divided into five groups: a non-diabetic untreated control group (SD, n = 8), an untreated control group (SDT-Cont, n = 8), an exercise group (SDT-Ex, n = 7), an empagliflozin treatment group (SDT-EMPA, n = 8), and an exercise plus empagliflozin treatment group (SDT-EMPA+Ex, n = 6). Data are presented as box-and-whisker plots, showing the median, interquartile range, and minimum and maximum values, with individual data points overlaid. Normally distributed data (p-ULK1/ULK1) were analyzed using one-way ANOVA followed by Tukey’s post hoc test, whereas non-normally distributed data (ULK1/α-tubulin) were analyzed using the Kruskal–Wallis test followed by the Steel–Dwass post hoc test. Although parametric analyses were applied, p-ULK1/ULK1 data are presented as medians in the figure to align with the data reporting in the manuscript, and mean ± standard error of the mean (SEM) values for normally distributed data are provided in Supplementary Table S2. ^#^*p* < 0.05 vs. SDT-Cont group.

Phosphorylation of ULK1, an indicator of autophagy initiation, was significantly higher in the SDT-EMPA + Ex group than in the SDT-Cont group, whereas no significant differences were found among the other groups (Fig. 6A, B). Total ULK1 protein expression did not differ significantly among any of the groups (Fig. 6A, C).

**Fig. 7:**
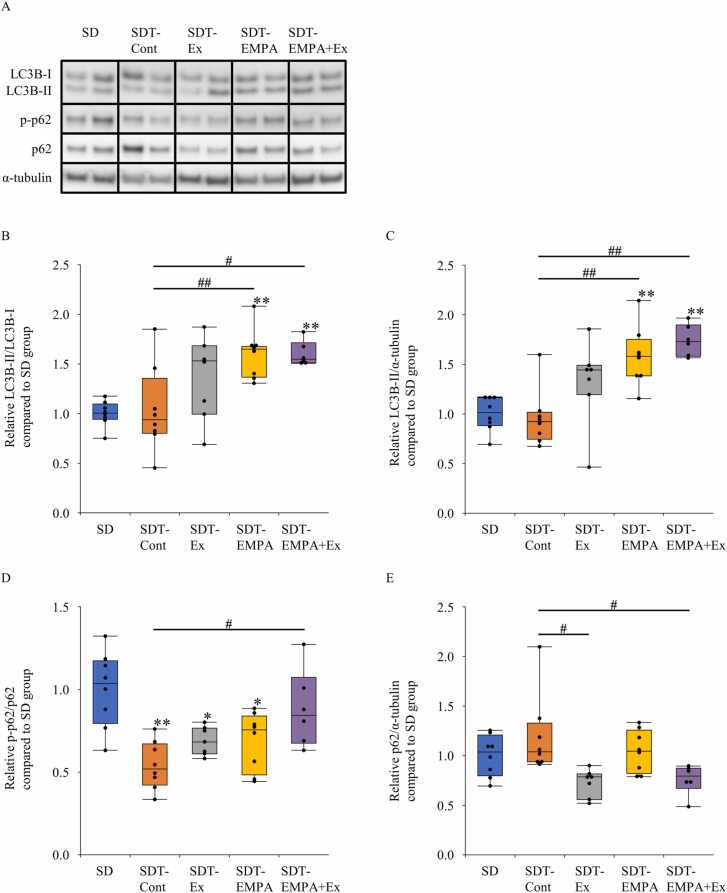
**Assessment of autophagy-related signaling in the soleus muscle by Western blotting.** Representative Western blotting bands of LC3B-I, LC3B-II, phosphorylated p62 (p-p62), total p62 (p62), and α-tubulin in each group on the same membrane (A). Samples from the same experiment were processed in parallel for SDS polyacrylamide gel electrophoresis (SDS-PAGE) and Western blotting using different gels and membranes, and the image data obtained were cropped. Entire Western blot images are shown in Supplementary Figs. S4. Quantification of protein expression: LC3B-II/LC3B-I (B), relative LC3B-II/α-tubulin (C), relative p-p62/p62 (D), and relative p62/α-tubulin (E), all normalized to the SD group. Rats were divided into five groups: a non-diabetic untreated control group (SD, n = 8), an untreated control group (SDT-Cont, n = 8), an exercise group (SDT-Ex, n = 7), an empagliflozin treatment group (SDT-EMPA, n = 8), and an exercise plus empagliflozin treatment group (SDT-EMPA+Ex, n = 6). Data are presented as box-and-whisker plots, showing the median, interquartile range, and minimum and maximum values, with individual data points overlaid. Normally distributed data (LC3B-II/LC3B-I, LC3B-II/α-tubulin, and p-p62/p62) were analyzed using one-way ANOVA followed by Tukey’s post hoc test, whereas non-normally distributed data (p62/α-tubulin) were analyzed using the Kruskal–Wallis test followed by the Steel–Dwass post hoc test. Although parametric analyses were applied, LC3B-II/LC3B-I, LC3B-II/α-tubulin, and p-p62/p62 data are presented as medians in the figure to align with the data reporting in the manuscript, and mean ± standard error of the mean (SEM) values for normally distributed data are provided in Supplementary Table S2. * *p* < 0.05 and ** *p* < 0.01 vs. SD group; ^#^*p* < 0.05 and ^##^*p* < 0.01 vs. SDT-Cont group.

The LC3B-II/LC3B-I ratio, a marker of autophagosome formation, was significantly elevated in the SDT-EMPA and SDT-EMPA + Ex groups compared with both the SD and SDT-Cont groups (Fig. 7A, B). A similar pattern was observed for total LC3B-II protein expression, which also reflects autophagosome content, and it was significantly higher in the SDT-EMPA and SDT-EMPA + Ex groups than in the SD and SDT-Cont groups (Fig. 7A, C).

Phosphorylation of p62, a marker associated with selective autophagy, was significantly reduced in the SDT-Cont, SDT-Ex, and SDT-EMPA groups compared with the SD group, whereas no significant difference was observed between the SD and SDT-EMPA + Ex groups. Additionally, p62 phosphorylation was significantly higher in the SDT-EMPA + Ex group than in the SDT-Cont group (Fig. 7A, D). Total p62 protein expression, an indicator of autophagic flux, was not different among the SD and SDT groups, although it was significantly lower in the SDT-Ex and SDT-EMPA + Ex groups than the SDT-Cont group (Fig. 7A, E).

## Discussion

In this study, we demonstrated that EMPA treatment preserved soleus muscle weight normalized to tibial length and the CSA of type I fibers in the soleus muscle, both of which were reduced in SDT fatty rats compared with SD rats under ad libitum feeding. Additionally, EMPA treatment upregulated PGC−1α and LC3B-II protein expression in the soleus muscle in SDT fatty rats. Moreover, combined EMPA and endurance exercise preserved the CSA of type I fibers in the soleus muscle of SDT fatty rats compared with SD rats. Meanwhile, combining EMPA with exercise significantly increased CS activity and the protein expression of PGC−1α, COX5B, phosphorylated ULK1, LC3B-II, and phosphorylated p62, accompanied by a reduction in total p62 protein expression, in the soleus muscle of SDT fatty rats. Collectively, these results suggest that EMPA treatment does not exacerbate skeletal muscle atrophy under ad libitum feeding conditions and may be associated with favorable changes in mitochondrial marker and autophagy-related processes in the soleus muscle of SDT fatty rats. Furthermore, when combined with exercise training, it may induce more pronounced improvements in mitochondrial content, mitochondrial biogenesis marker and the expression of specific respiratory chain protein, as well as in autophagic flux. Mitochondrial status and autophagic flux are critical for maintaining muscle quality. Clinical evidence suggests that lower mitochondrial capacity is directly associated with impaired mobility, such as slower walking speed [26], while defective autophagy leads to the loss of muscle integrity and mass [27]. Therefore, the improvements in these markers observed in the combined treatment group might translate to enhanced physical function and a reduced risk of falls in the clinical setting.

While the EMPA-ELDERLY trial reported that EMPA treatment decreased bodyweight in elderly patients [5], we observed an increase in body weight by EMPA treatment in SDT fatty rats. We considered this discrepancy to arise from the specific characteristics of our animal model and the absence of dietary intervention. Unlike clinical practice where diet restriction is standard, SDT fatty rats exhibit inherent hyperphagia due to leptin receptor abnormalities that impair satiety signaling [15]. This is consistent with previous findings that SGLT2 inhibitors reduce body weight in hyperphagic db/db mice only when combined with diet restriction [6]. Furthermore, SGLT2 inhibitors consistently decrease body weight in non-obese, non-hyperphagic models, such as streptozotocin-induced diabetic rats [7]. In clinical settings, while SGLT2 inhibitors typically reduce weight in T2DM patients with diet restriction, the preservation of muscle-related markers in our model may suggest a therapeutic potential for preventing the excessive catabolism associated with advanced T2DM. We speculate that our findings are particularly relevant to obese T2DM patients at high risk for sarcopenic obesity, where preserving muscle quality while managing metabolic status is a priority.

A previous study using the SGLT2 inhibitor dapagliflozin also reported body weight gain in SDT fatty rats [19], a finding supported by our present study. EMPA treatment alone led to a significant increase in body weight at 12 and 16 weeks of age compared to the respective non-EMPA groups. Regarding the composition of this weight gain, several factors must be considered. First, although food intake was significantly higher in all SDT fatty rat groups compared to SD rats, there were no differences among the SDT fatty groups, suggesting that additional hyperphagia was not the primary driver of the EMPA-induced weight increase. Second, regarding the possibility of fluid retention; urine volume in the SDT fatty groups exceeded water intake (Supplementary Table S3), and this negative fluid balance—driven by osmotic diuresis—effectively rules out edema. Importantly, the EMPA-treated groups showed a reduction in adipose tissue weight normalized to body weight, indicating that the weight gain is unlikely to reflect increased adiposity. In our previous study [18], untreated SDT fatty rats eventually exhibited lower body weight than SD rats at 20–24 weeks as diabetes progressed toward a severely catabolic state. In the present study, EMPA treatment alleviated severe hyperglycemia and improved HOMA-IR, likely shifting the metabolic state from catabolism to anabolism. While the increased weights of the soleus and extensor digitorum longus muscles (Supplementary Table S4) do not fully account for the total body weight increase, our data suggest that EMPA treatment promotes tissue maintenance and qualitative improvements in body composition rather than fat accumulation. However, as body composition was not directly assessed using DXA or MRI, further investigation is required to fully elucidate these underlying mechanisms.

In obese T2DM, insulin resistance and dyslipidemia contribute to skeletal muscle abnormalities [28]. A recent clinical study reported that dyslipidemia in patients with T2DM was a significant risk factor for sarcopenia [9]. Thus, targeting dyslipidemia in addition to insulin resistance may help ameliorate skeletal muscle frailty. Both EMPA treatment alone and in combination with exercise similarly improved hyperglycemia and insulin resistance relative to untreated SDT fatty rats. Furthermore, combination therapy improved dyslipidemia more than EMPA treatment alone, an effect comparable to that observed with exercise training alone. Therefore, although caloric intake was reduced by SGLT2 inhibitor–induced urinary glucose excretion and caloric expenditure was increased by exercise under combined EMPA and exercise treatment, a further reduction in grip strength, muscle weight, and CSA of type I fibers was not induced in the soleus compared with EMPA treatment alone. Moreover, combining EMPA and exercise training increased the expression of several proteins associated with mitochondrial function and enhanced autophagic flux compared with EMPA treatment alone in the soleus muscle of SDT fatty rats. Since the soleus muscle predominantly consists of oxidative muscle fibers with high fatty acid metabolic capacity [29], improving dyslipidemia may mitigate lipotoxicity, creating a metabolic environment that facilitates mitochondrial function restoration and autophagy activation [30], [31]. Taken together, combining EMPA and exercise training, which improved both insulin resistance and dyslipidemia, may impart more favorable effects on the soleus muscle than EMPA treatment alone, which improved insulin resistance but not dyslipidemia in SDT fatty rats.

Accumulating evidence indicates that impaired autophagic flux and p62 accumulation contribute to skeletal muscle dysfunction in obese T2DM, with oxidative muscles like the soleus being particularly susceptible [32], [33], [34], [35]. Autophagy dysregulation is an important pathological mechanism linking insulin resistance and muscle atrophy. Previous studies have shown that SGLT2 inhibitors, including EMPA, activate autophagy-related signaling pathways, as evidenced by increased LC3B-II expression in various tissues, including the kidney, heart, and other organs regardless of their SGLT2 expression levels [12], [13], [14]. These findings suggest that EMPA could stimulate autophagosome formation through systemic, SGLT2-independent mechanisms, raising the possibility that similar autophagy-promoting effects could occur in skeletal muscle. In contrast, exercise has been shown to improve autophagic flux in skeletal muscle, as evidenced by increased lysosomal activity and reduced p62 protein expression, indicating more efficient autophagosome clearance [36], [37], [38]. In this study, EMPA treatment alone increased LC3B-II expression, whereas combined EMPA and exercise training was associated with both increased phosphorylation of p62 and reduced total p62 protein expression, suggesting that exercise may complement EMPA-induced autophagosome formation by promoting autophagic flux. Therefore, the combination of EMPA and exercise training may result in more effective activation of autophagy than EMPA treatment alone in the soleus muscle of SDT fatty rats. Importantly, enhanced autophagy in skeletal muscle has been reported to contribute to the maintenance of mitochondrial quality and function, in part through the selective removal of damaged mitochondria (mitophagy) [37], [38]. Thus, the combined intervention may promote autophagic flux, underpinning favorable changes in mitochondrial status and oxidative muscle fiber integrity.

Although muscle weight and the CSA of type I fibers in the soleus were significantly lower in untreated SDT fatty rats compared with SD rats, the expression of several proteins associated with mitochondrial function and autophagic flux was similar between the two groups, suggesting that these proteins may not directly contribute to soleus muscle atrophy. Moreover, although the effects of EMPA treatment alone or in combination with exercise training affected mitochondrial status and autophagic flux, these changes did not translate into improvements in muscle strength. Further studies are needed to evaluate the effects of EMPA treatment alone or combined with exercise training in models in which mitochondrial abnormalities and impaired autophagic flux are causally involved in soleus muscle atrophy. Moreover, the treadmill exercise protocol used in this study was mild, as exercise alone did not increase CS activity or alter the expression of proteins related to mitochondrial function compared with untreated rats. Additionally, although the treadmill running speed used in this study falls within the moderate intensity range, the overall exercise stimulus, including exercise duration and frequency, may have been modest, as exercise alone did not increase CS activity or alter the expression of proteins related to mitochondrial function compared with untreated rats. Furthermore, the treadmill exercise protocol in this study primarily represents aerobic exercise, which may have limited effects on muscle strength, particularly when compared with resistance exercise. Further studies are warranted to determine whether different exercise intensities or types are more effective at enhancing muscle strength.

As representative markers of the respiratory chain, the expression of COX4 and COX5B was measured. While the COX4 protein expression did not show significant differences among the groups (Supplementary Figure S1), a significant increase in the COX5B expression was observed in the combined EMPA and exercise group compared with the untreated or exercise-alone groups. Our previous study using the same disease model (SDT fatty rats), which demonstrated that glucagon-like peptide−1 receptor agonist (liraglutide) treatment significantly modulated both CS activity and COX5B protein expression in the soleus muscle [21]. While COX4 and COX5B are both subunits of Complex IV, their expression patterns can dissociate depending on the physiological stimulus. The significant change observed only in COX5B suggests that our intervention may have a subunit-specific impact on the mitochondrial proteome, or that COX5B is a more sensitive marker for this particular disease model. Further studies are needed to clarify the precise mechanisms underlying these subunit-specific responses.

The stimulatory effects of exercise on mitochondrial regulation and the PGC−1α pathway are indeed well established. However, a key finding of our study is that in this specific severe obesity and diabetes, exercise training alone was insufficient to significantly increase mitochondrial markers compared to the sedentary control. Crucially, we found that the combination of EMPA and exercise led to significant improvements in PGC−1α and COX5B expression compared to both the sedentary control and the exercise-alone groups. While this effect was not observed across all measured proteins (e.g., COX4), the significant modulation of CS activity and COX5B expression suggests that the combination therapy at least partially enhances mitochondrial protein profiles under conditions where exercise training alone failed. This provides a novel clinical insight: for individuals with severe metabolic impairment who may not respond to moderate exercise alone, the addition of EMPA may potentiate or support the manifestation of exercise-induced adaptations in certain mitochondrial pathways. Thus, EMPA might serve not only as a glucose-lowering agent but also as a potential "exercise-sensitizer" that enhances the physiological benefits of physical activity in the context of sarcopenic obesity.

This study has several limitations. First, pathophysiological analysis of the soleus muscle was performed only in SDT fatty and SD rats at 16 weeks old. Additionally, the relationship between changes in pathophysiology from 8 to 16 weeks of age and the effects of interventions was not evaluated in each group. Second, this study did not include measures of physical performance, such as gait speed or physical coordination, due to the characteristically low levels of spontaneous activity in SDT fatty rats. Since the clinical diagnosis of sarcopenia requires a combination of muscle mass, strength, and physical performance, the lack of functional data is a limitation. Third, EMPA administration and/or exercise were not performed in SD rats as nondiabetic controls because SGLT2 inhibitors are expected to have minimal effects under normoglycemic conditions, and SD rats do not exhibit skeletal muscle atrophy. Therefore, whether the observed effect was specific to obese T2DM pathology remains unclear. Fourth, this study was conducted under ad libitum feeding conditions. Therefore, it is uncertain whether similar effects would be observed under caloric restriction. Finally, direct whole-body imaging, such as DXA or MRI, was not performed to precisely quantify changes in body composition, including lean and fat mass. Future studies using diverse models and functional assessments are warranted to further validate these findings in the context of clinical sarcopenia.

In conclusion, in the soleus muscle in a rat model of T2DM, EMPA treatment does not exert detrimental effects and may induce favorable changes in mitochondrial marker and autophagy-related processes. Furthermore, combining exercise training with EMPA may enhance mitochondrial status and autophagic flux in the soleus muscle compared with EMPA treatment alone, in part by improving insulin resistance and dyslipidemia.

## Ethics approval and consent to participate

All animal experiments were performed following the ethical standards of St. Marianna University School of Medicine. The experimental protocol was approved by the Animal Experiment Committee at the Institute for Animal Experimentation, St. Marianna University Graduate School of Medicine (Approval No. 2406001).

## Funding

This study was conducted as a collaborative research project and was financially supported by Nippon Boehringer Ingelheim Co., Ltd (the manufacturer of empagliflozin).

## CRediT authorship contribution statement

**Kazuho Inoue:** Writing – review & editing, Writing – original draft, Visualization, Validation, Supervision, Software, Project administration, Methodology, Investigation, Formal analysis, Data curation, Conceptualization. **Saori Sekiguchi:** Writing – review & editing, Validation, Supervision, Resources, Project administration, Methodology, Conceptualization. **Yuji Ogura:** Writing – review & editing, Validation, Supervision, Resources, Project administration, Methodology, Conceptualization. **Seiko Hoshino:** Investigation, Data curation. **Kimie Katayama:** Investigation, Data curation. **Junko Asano:** Investigation, Data curation. **Takayuki Akagi:** Investigation, Data curation. **Junko Igarashi-Migitaka:** Investigation, Data curation. **Shiika Watanabe:** Investigation, Data curation. **Yoshio Nagai:** Writing – review & editing, Validation, Supervision. **Kenjiro Kimura:** Writing – review & editing, Validation. **Yugo Shibagaki:** Writing – review & editing, Validation, Supervision, Project administration, Methodology, Funding acquisition, Conceptualization. **Atsuko Kamijo-Ikemori:** Writing – review & editing, Writing – original draft, Visualization, Validation, Supervision, Software, Resources, Project administration, Methodology, Investigation, Funding acquisition, Formal analysis, Data curation, Conceptualization.

## Declaration of Competing Interest

The authors declare the following financial interests/personal relationships which may be considered as potential competing interests: Yugo Shibagaki reports financial support and equipment, drugs, or supplies were provided by Nippon Boehringer Ingelheim Co Ltd. Saori Sekiguchi and Yuji Ogura are employees at Nippon Boehringer Ingelheim Co., Ltd. All other authors declare no conflicts of interest. If there are other authors, they declare that they have no known competing financial interests or personal relationships that could have appeared to influence the work reported in this paper.

## Data Availability

The data used to support the findings of this study are included within the article.

## Glossary

CSA: Cross-sectional area
CS: Citrate synthase
EMPA: Empagliflozin
HOMA-IR: Homeostasis Model Assessment of Insulin Resistance
LC3B: Microtubule-associated protein 1 light chain 3 beta
MCAD: Medium-chain acyl-CoA dehydrogenase
PGC-1α: Peroxisome proliferator-activated receptor gamma coactivator-1 alpha
p62 / SQSTM1: Sequestosome 1
ULK1: Unc-51-like kinase 1
SGLT2: Sodium-glucose cotransporter 2
SD: Sprague–Dawley
SDT: Spontaneously diabetic Torii
T2DM: Type 2 diabetes mellitus

## Footnotes

Appendix A Supplementary data associated with this article can be found in the online version at doi:10.1016/j.jphyss.2026.100087.
