# Supplementary material for: Effects of empagliflozin alone and in combination with exercise on the soleus muscle in obese type 2 diabetic rats
Source: J Physiol Sci. 2026 Jul 7;76(3):100087. doi: 10.1016/j.jphyss.2026.100087 (PMC13351120; doi:10.1016/j.jphyss.2026.100087)
Supplement: Supplementary file 1 — Supplementary material [file mmc1.pdf]

Effects of Empagliflozin Alone and in Combination with Exercise on Soleus Muscle in Obese Type 2 Diabetic Rats

Kazuho Inoue[1], Saori Sekiguchi[2], Yuji Ogura[2], Seiko Hoshino[1], Kimie Katayama[3], Junko Asano[3], Takayuki Akagi[1], Junko Migitaka[1], Shiika Watanabe[3], Yoshio Nagai[4], Kenjiro Kimura[3, 5], Yugo Shibagaki[3], Atsuko Kamijo-Ikemori[1, 3, 6, *]

1. Department of Anatomy, St. Marianna University School of Medicine, Kanagawa, Japan.

2. Medicine Division, Nippon Boehringer Ingelheim Co., Ltd., Tokyo, Japan.

3. Division of Nephrology and Hypertension, Department of Internal Medicine, St. Marianna University School of Medicine, Kanagawa, Japan.

4. Department of Diabetes and Endocrinology, Kanto Rosai Hospital, Kanagawa, Japan.

5. JCHO Tokyo Takanawa Hospital, Tokyo, Japan.

6. Institute for Animal Experimentation, St. Marianna University Graduate School of Medicine, Kanagawa, Japan.

*Corresponding author

Corresponding author: Atsuko Kamijo-Ikemori, M.D., Ph.D.

Department of Anatomy, and Division of Nephrology and Hypertension, Department of Internal Medicine, St. Marianna University School of Medicine

2-16-1 Sugao, Miyamae-Ku, Kawasaki 216-8511, Japan

Tel: +81-44-977-8111 ext. 3630, Fax: +81-44-976-7083, E-mail: a2kamijo@marianna-u.ac.jp

Supplementary *Tables*

Supplementary Table S1. Shapiro–Wilk test for evaluating whether each variable follows a normal distribution

Variable	Shapiro–Wilk test statistic (W)	p-value	Normal / Non-normal
Body weight (g)	0.9513323	0.0002	Non-normal
Food intake (g)	0.9250186	<0.0001	Non-normal
Water intake (g)	0.940551	<0.0001	Non-normal
Grip strength/body weight (N/kg)	0.8647124	<0.0001	Non-normal
Blood glucose level (mg/dL)	0.8868005	<0.0001	Non-normal
Fasting blood glucose level (mg/dL)	0.8652566	0.0004	Non-normal
Fasting serum insulin level (μIU/mL)	0.6258762	<0.0001	Non-normal
Homeostasis Model Assessment of Insulin Resistance	0.6914066	<0.0001	Non-normal
Total cholesterol level (mg/dL)	0.943104	0.0579	Normal
Triglyceride levelTriglyceride level (mg/dL)	0.9327519	0.0273	Non-normal
Combined weight of peritesticular and perirenal adipose tissue (g)	0.8425678	<0.0001	Non-normal
Combined weight of peritesticular and perirenal adipose tissue (% body weight)	0.8157781	<0.0001	Non-normal
Soleus muscle weight (g)	0.8516721	<0.0001	Non-normal
Soleus muscle weight normalized to tibial length (mg/mm)	0.8764835	0.0005	Non-normal
Cross-sectional area of type I fibers in the soleus (μm^2)	0.9908157	0.985	Normal
Citrate synthase activity in the soleus ($mM \cdot min^{-1} \cdot mg\ protein^{-1}$)	0.9618201	0.2306	Normal
Relative PGC-1α/α-tubulin compared to SD group	0.6606802	<0.0001	Non-normal
Relative MCAD/α-tubulin compared to SD group	0.6674811	<0.0001	Non-normal
Relative COX5B/α-tubulin compared to SD group	0.9724891	0.4799	Normal
Relative p-ULK1/ULK1 compared to SD group	0.9592509	0.1913	Normal
Relative ULK1/α-tubulin compared to SD group	0.9338615	0.0295	Non-normal
Relative LC3B-II/LC3B-I compared to SD group	0.9658727	0.3079	Normal
Relative LC3B-II/α-tubulin compared to SD group	0.980298	0.7418	Normal
Relative p-p62/p62 compared to SD group	0.9624657	0.2416	Normal
Relative p62/α-tubulin compared to SD group	0.8884994	0.0014	Non-normal

Shapiro–Wilk test results for all measured variables. The column "Shapiro–Wilk test statistic (W)" shows the test statistic, and the "p-value" column indicates statistical significance. Variables with $p \geq 0.05$ were classified as normally distributed, while those with $p < 0.05$ were classified as non-normal.

Supplementary Table S2. Mean ± standard errors of the mean (SEM) of variables analyzed using parametric statistical methods

Parameter	Group	16 weeks of age
Total cholesterol level (mg/dL)	SD	68.0 ± 3.5
	SDT-Cont	160.1 ± 9.3 **
	SDT-Ex	135.7 ± 8.1 **
	SDT-EMPA	167.8 ± 7.1 **, †
	SDT-EMPA+Ex	132.8 ± 7.5 **, §
Cross-sectional area of type I fibers in the soleus (μm^2)	SD	3709 ± 192
	SDT-Cont	3063 ± 110 *
	SDT-Ex	3124 ± 168
	SDT-EMPA	3216 ± 153
	SDT-EMPA+Ex	3442 ± 99
Citrate synthase activity in the soleus ($mM \cdot min^{-1} \cdot mg\ protein^{-1}$)	SD	0.233 ± 0.010
	SDT-Cont	0.228 ± 0.016
	SDT-Ex	0.258 ± 0.007
	SDT-EMPA	0.250 ± 0.020
	SDT-EMPA+Ex	0.311 ± 0.022 *, #
Relative COX5B/α-tubulin compared to SD group	SD	1.00 ± 0.14
	SDT-Cont	0.72 ± 0.09
	SDT-Ex	0.80 ± 0.05
	SDT-EMPA	1.18 ± 0.10
	SDT-EMPA+Ex	1.35 ± 0.20 ##, †
Relative p-ULK1/ULK1 compared to SD group	SD	1.00 ± 0.04
	SDT-Cont	0.91 ± 0.07
	SDT-Ex	1.11 ± 0.04
	SDT-EMPA	1.11 ± 0.07
	SDT-EMPA+Ex	1.19 ± 0.08 #

Relative LC3B-II/LC3B-I compared to SD group	SD	1.00 ± 0.05
	SDT-Cont	1.04 ± 0.15
	SDT-Ex	1.35 ± 0.16
	SDT-EMPA	1.60 ± 0.09 **, ##
	SDT-EMPA+Ex	1.60 ± 0.05 **, #
Relative LC3B-II/α-tubulin compared to SD group	SD	1.00 ± 0.06
	SDT-Cont	0.96 ± 0.10
	SDT-Ex	1.32 ± 0.16
	SDT-EMPA	1.58 ± 0.11 **, ##
	SDT-EMPA+Ex	1.74 ± 0.06 **, ##
Relative p-p62/p62 compared to SD group	SD	1.00 ± 0.08
	SDT-Cont	0.54 ± 0.05 **
	SDT-Ex	0.69 ± 0.03 *
	SDT-EMPA	0.69 ± 0.06 *
	SDT-EMPA+Ex	0.88 ± 0.10 #

Mean values and SEM are shown for variables that were classified as normally distributed based on the Shapiro–Wilk test (see Supplementary Table S1). These variables were analyzed using parametric statistical methods. Comparisons among the five groups were performed using one-way analysis of variance (ANOVA) followed by Tukey's honestly significant difference (HSD) post hoc test. Rats were divided into five groups: a non-diabetic untreated control group (SD, n = 8), an untreated control group (SDT-Cont, n = 8), an exercise group (SDT-Ex, n = 7), an empagliflozin treatment group (SDT-EMPA, n = 8), and an empagliflozin plus exercise treatment group (SDT-EMPA+Ex, n = 6). * $p < 0.05$ and ** $p < 0.01$ vs. SD group; # $p < 0.05$ and ## $p < 0.01$ vs. SDT-Cont group; † $p < 0.05$ vs. SDT-Ex group; § $p < 0.05$ vs. SDT-EMPA group.

Supplementary Table S3. Time-related changes in urinary volume

Parameter	Group	8 weeks of age	12 weeks of age	16 weeks of age
Urinary volume (ml)	SD	12.5 (6.5–28.6)	20.8 (11.0–25.2)	13.5 (6.5–36.2)
	SDT-Cont	12.1 (8.6–29.3)	27.7 (13.5–45.0) ‡	25.1 (6.8–55.4) ‡‡
	SDT-Ex	15.1 (8.8–36.6)	18.6 (17.0–28.8)	27.2 (11.5–41.0)
	SDT-EMPA	11.1 (3.0–27.6)	34.7 (23.2–45.4) **, ††, ‡‡	35.5 (21.2–55.0) *, ‡‡
	SDT-EMPA+Ex	14.5 (11.3–20.2)	25.9 (21.0–28.0)	25.6 (24.0–37.0) †, ‡

All data are presented as medians with ranges. The normality of the data distribution in urinary volume was assessed using the Shapiro–Wilk test, which indicated non-normal distributions for 8 weeks of age (W = 0.8998, p = 0.0016), and normal distributions for both 12 weeks of age (W = 0.9504, p = 0.0721) and 16 weeks of age (W = 0.9631, p = 0.2265). Accordingly, non-normally distributed data was analyzed using the Kruskal–Wallis test followed by the Steel–Dwass post hoc test, whereas normally distributed data were analyzed using one-way ANOVA followed by Tukey's post hoc test. Although parametric analyses were applied, data at 12 and 16 weeks of age are presented as medians in the figure to align with the data reporting in the manuscript; however, mean ± standard error of the mean values for normally distributed data are as follows: at 12 weeks of age, SD (20.2 ± 1.7), SDT-Cont (28.9 ± 3.4), SDT-Ex (19.7 ± 1.6), SDT-EMPA (34.5 ± 2.3), and SDT-EMPA+Ex (25.3 ± 1.1); at 16 weeks of age, SD (16.5 ± 3.5), SDT-Cont (27.6 ± 5.2), SDT-Ex (27.3 ± 4.0), SDT-EMPA (34.6 ± 4.0), and SDT-EMPA+Ex (28.1 ± 2.2). For longitudinal comparisons within the same group across multiple time points, differences over time were analyzed using the Friedman rank test, followed by the Nemenyi post hoc test when a significant main effect was detected. Rats were divided into five groups: a non-diabetic untreated control group (SD, n = 8), an untreated control group (SDT-Cont, n = 8), an exercise group (SDT-Ex, n = 7), an empagliflozin treatment group (SDT-EMPA, n = 8), and an empagliflozin plus exercise treatment group (SDT-EMPA+Ex, n = 6). * $p < 0.05$ and ** $p < 0.01$ vs. SD group at the same age; †† $p < 0.01$ vs. SDT-Ex group at the same age; ‡ $p < 0.05$ and ‡‡ $p < 0.01$ vs. the same group at 8 weeks old.

Supplementary Table S4. Extensor digitorum longus (EDL) muscle weight and tibial length–normalized EDL muscle weight

Parameter	Group	16 weeks of age
EDL muscle weight (g)	SD	0.507 (0.453–0.578)
	SDT-Cont	0.329 (0.266–0.363) **
	SDT-Ex	0.327 (0.301–0.352) *
	SDT-EMPA	0.371 (0.352–0.405) $^{**, \#, \dagger}$
	SDT-EMPA+Ex	0.368 (0.305–0.377) *
EDL muscle weight normalized to tibial length (mg/mm)	SD	11.46 (10.40–13.15)
	SDT-Cont	8.27 (6.93–8.93) **
	SDT-Ex	8.30 (7.59–8.89) *
	SDT-EMPA	9.13 (8.86–10.08) $^{**, \#}$
	SDT-EMPA+Ex	9.26 (7.94–9.42) *

All data are presented as medians with ranges. The normality of the data distribution was assessed using the Shapiro–Wilk test, which indicated non-normal distributions for both EDL muscle weight (W = 0.8678, p = 0.0004) and EDL muscle weight normalized to tibial length (W = 0.9147, p = 0.0068). Accordingly, non-normally distributed data were analyzed using the Kruskal–Wallis test followed by the Steel–Dwass post hoc test. Rats were divided into five groups: a non-diabetic untreated control group (SD, n = 8), an untreated control group (SDT-Cont, n = 8), an exercise group (SDT-Ex, n = 7), an empagliflozin treatment group (SDT-EMPA, n = 8), and an empagliflozin plus exercise treatment group (SDT-EMPA+Ex, n = 6). * $p < 0.05$ and ** $p < 0.01$ vs. SD group; $^{\#}p < 0.05$ and $^{\#\#}p < 0.01$ vs. SDT-Cont group; $^{\dagger}p < 0.05$ vs. SDT-Ex group; $^{\S}p < 0.05$ vs. SDT-EMPA group.

Supplementary Figures

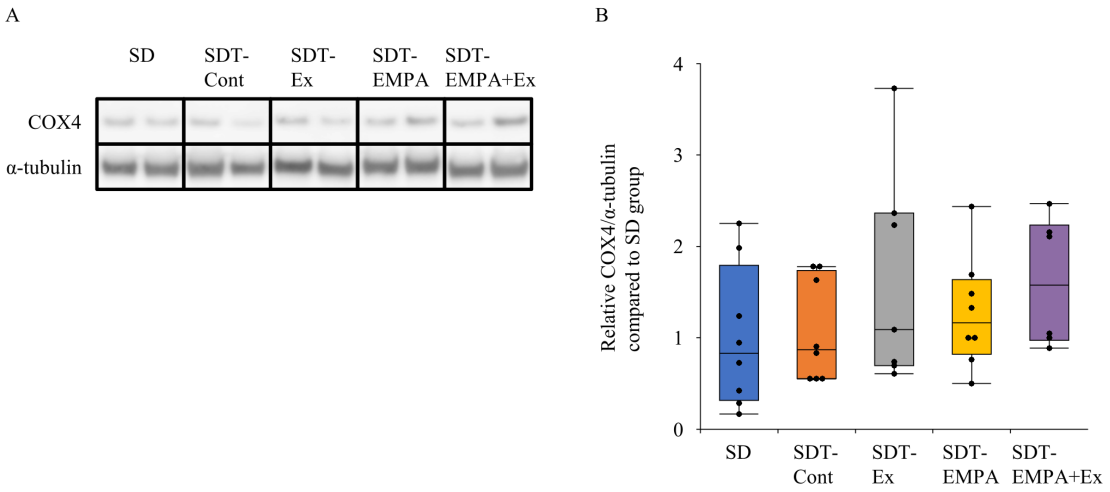

Supplementary Figure S1. Quantification of COX4 in the soleus muscle by Western blotting. Representative Western blotting bands of COX4 and α-tubulin in each group on the same membrane (A). COX4 protein was detected using the anti-COX4 antibody (ab202554, Abcam) by the same Western blotting protocol as described in the Methods section, and α-tubulin was detected using the same antibody as described in the Methods section. Samples from the same experiment were processed in parallel for SDS polyacrylamide gel electrophoresis (SDS-PAGE) and Western blotting using different gels and membranes, and the image data obtained were cropped. Entire Western blot images are shown in Supplementary Figures S5. Quantification of protein expression relative to α-tubulin and normalized to the SD group: COX4/α-tubulin (B). Rats were divided into five groups: a non-diabetic untreated control group (SD, n = 8), an untreated control group (SDT-Cont, n = 8), an exercise group (SDT-Ex, n = 7), an empagliflozin treatment group (SDT-EMPA, n = 8), and an exercise plus empagliflozin treatment group (SDT-EMPA+Ex, n = 6). Data are presented as box-and-whisker plots, showing the median, interquartile range, and minimum and maximum values, with individual data points overlaid. The normality of the data distribution was assessed using the Shapiro–Wilk test, which indicated non-normal distributions for COX4/α-tubulin (W = 0.918623, p = 0.0124). Accordingly, non-normally distributed data were analyzed using the Kruskal–Wallis test followed by the Steel–Dwass post hoc test.

No significant differences in COX4/α-tubulin ratio were observed among groups.

Entire images of Western Blotting

Supplementary Figure S2

A

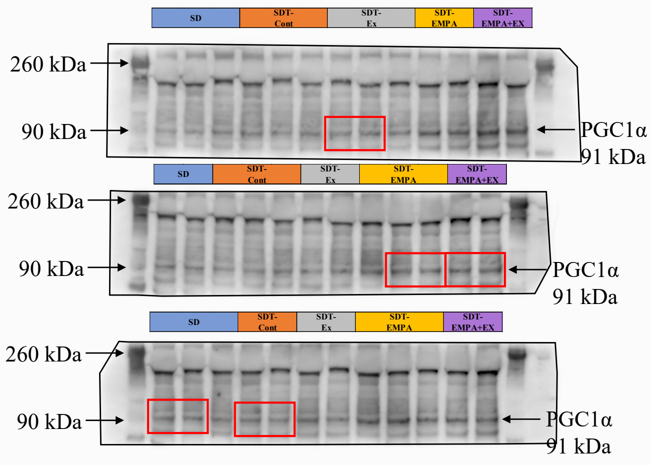

B

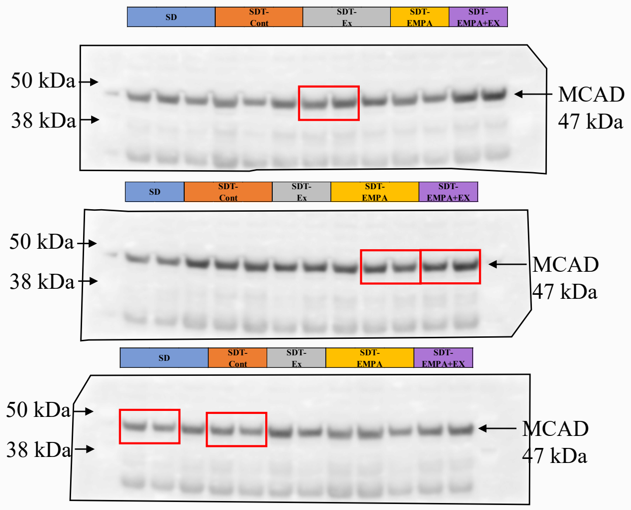

C

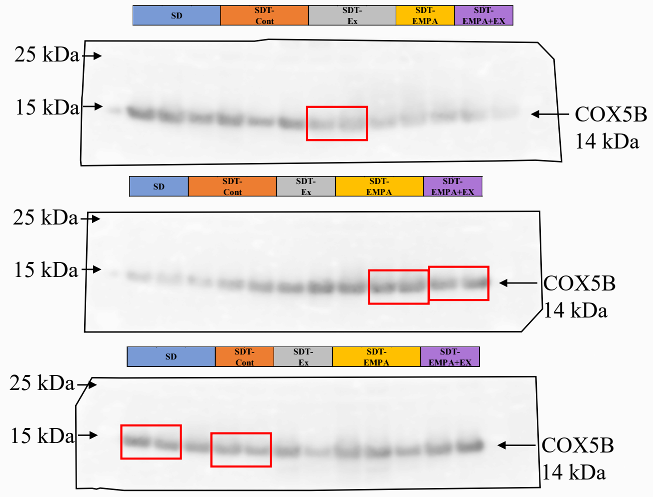

D

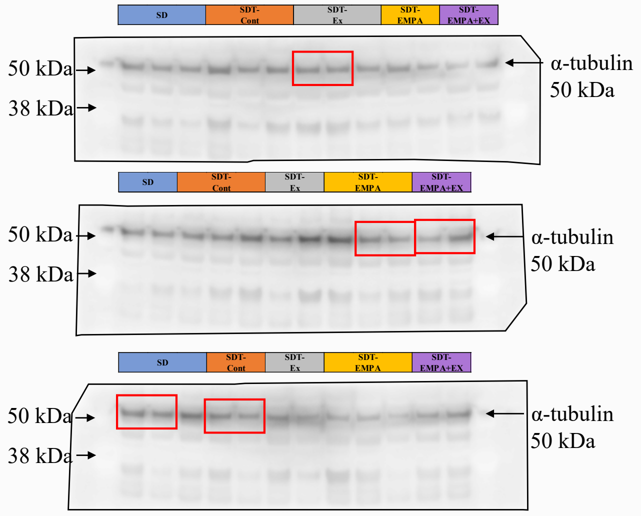

Western blotting of PGC1α (A), MCAD (B), COX5B (C), and α-Tubulin (D, used as a loading control) are shown. Black lines indicate the edges of each cut membrane, and red boxes highlight the regions of the original blots used in the main figures.

Supplementary Figure S3

A

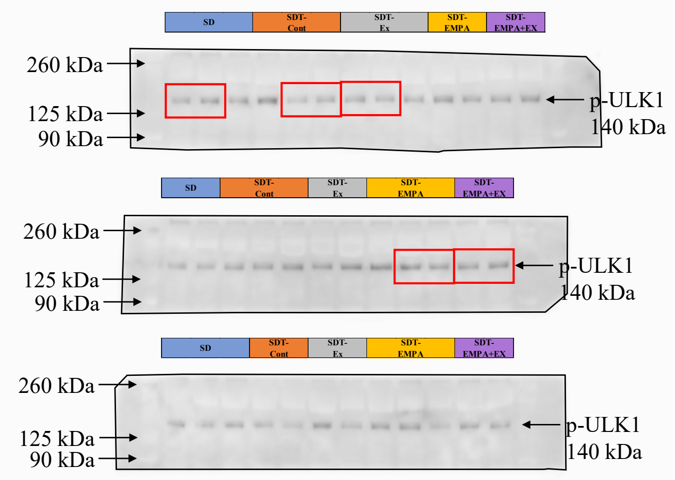

B

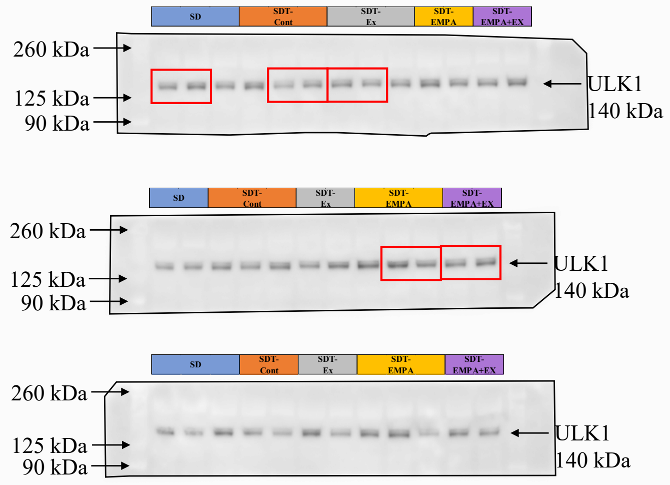

C

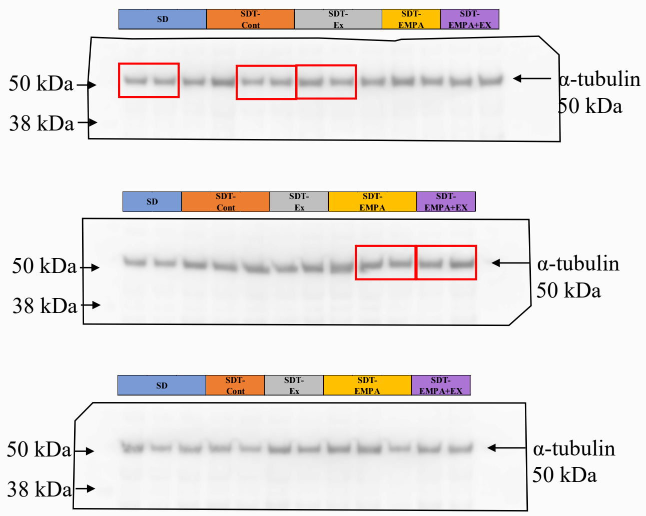

Western blotting of phosphorylated ULK1 (A, p-ULK1), total ULK1 (B, ULK1), and α-Tubulin (C, used as a loading control) are shown. Black lines indicate the edges of each cut membrane, and red boxes highlight the regions of the original blots used in the main figures.

Supplementary Figure S4

A

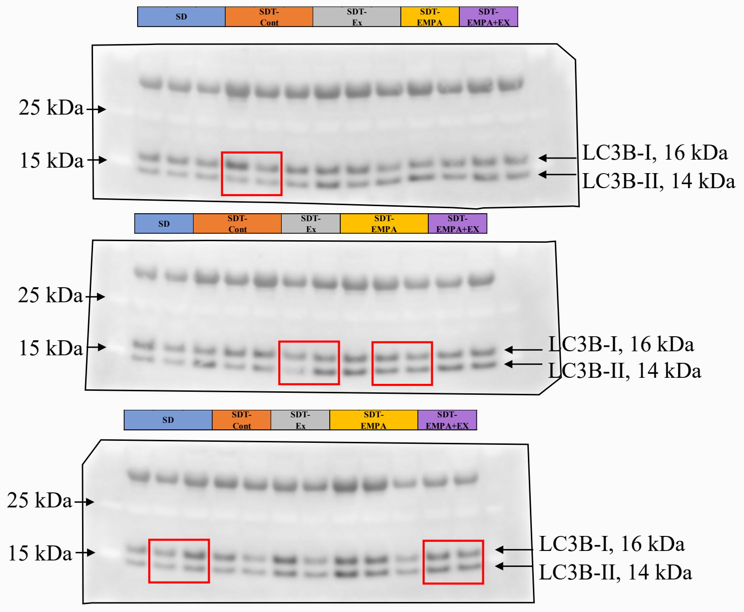

B

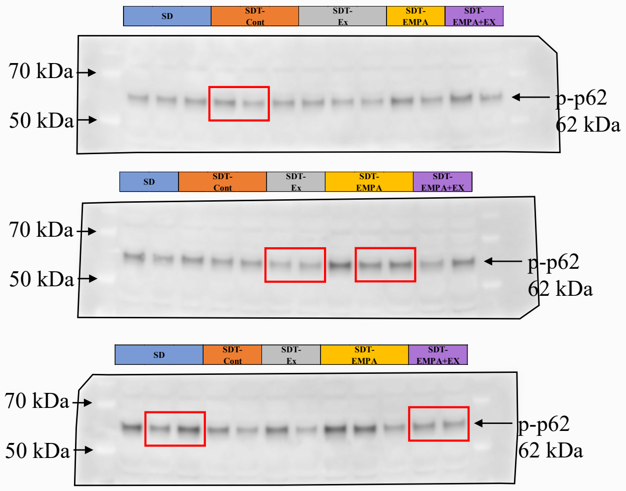

C

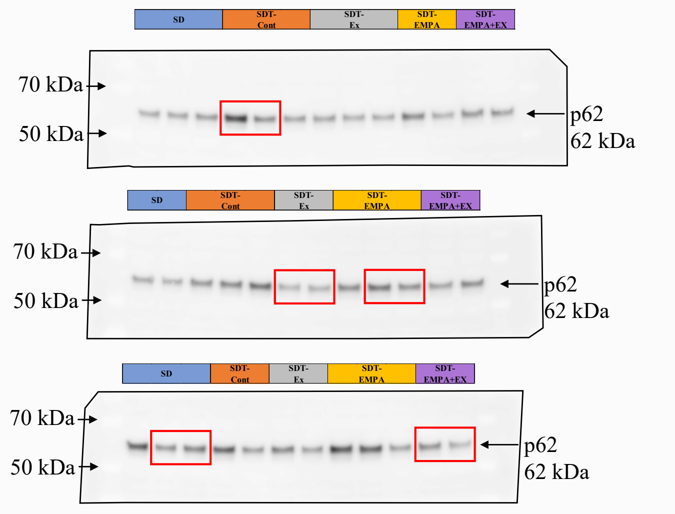

D

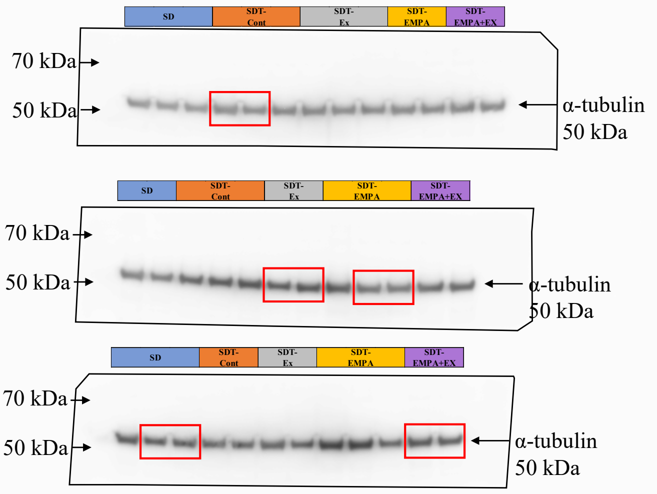

Western blotting of LC3B (A, LC3B-I and LC3B-II), phosphorylated p62 (B, p-p62), total p62 (C, p62), and α-Tubulin (D, used as a loading control) are shown. Black lines indicate the edges of each cut membrane, and red boxes highlight the regions of the original blots used in the main figures.

Supplementary Figure S5

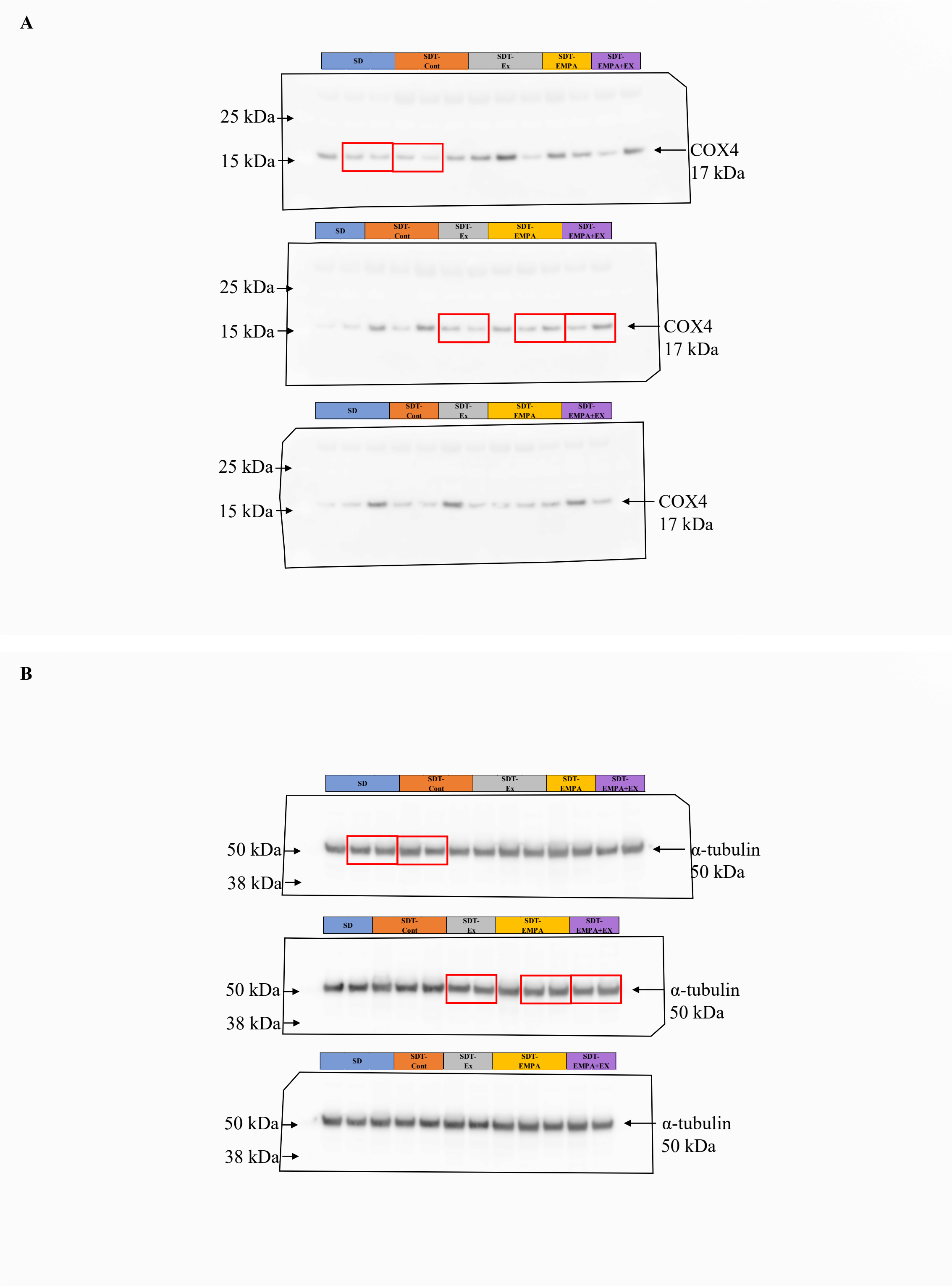

Western blotting of COX4 (A) and α-Tubulin (B, used as a loading control) are shown. Black lines indicate the edges of each cut membrane, and red boxes highlight the regions of the original blots used in the Supplementary Figures S1.
